# American Medical Association—Section on Stomatology

**Published:** 1898-07

**Authors:** 


### American Medical Association—Section on Stomatology.

The annual meeting of the American Medical Association was held at Denver, Colorado, June 7th to 10th. All things considered, it was a very large, successful and interesting meeting.

There were over 1,500 members present. Report of the work done in the sections was very encouraging indeed. Many important papers were read and discussed.

The section in which the dentists were more particularly interested was that of Stomatology. The Section was quite encouraged in having a joint meeting with the Colorado State Society, making an attendance in the joint meeting about 100

members. A number of excellent papers were read and discussed. These will appear in the journal of the American Medical Association and in dental journals in due time.

Pertinent papers were read in this section by physicians from other sections and members of this section read papers in other sections, especially the surgical and neurological, thus creating a helpfulness between physicians and dentists.

The Association will meet next year in Columbus, Ohio, when it is to be hoped there will be a very much larger attendance upon the Stomatological Section than ever before, this being the most central point at which the Association has been held for years. We trust that the prominent members of the dental profession will keep this matter in mind and be prepared to attend that meeting.

